# Monoamine alterations in Alzheimer’s disease and their implications in comorbid neuropsychiatric symptoms

**DOI:** 10.1007/s11357-024-01359-x

**Published:** 2024-09-27

**Authors:** Shalini Saggu, Ava Bai, Mae Aida, Hasibur Rehman, Andrew Pless, Destany Ware, Ferenc Deak, Kai Jiao, Qin Wang

**Affiliations:** 1 Department of Neuroscience and Regenerative Medicine, Medical College of Georgia at Augusta University, Augusta, GA 30912 USA; 2 Center for Biotechnology and Genomic Medicine, Medical College of Georgia at Augusta University, Augusta, GA 30912 USA

**Keywords:** Alzheimer’s disease, Neuropsychiatric symptoms, Monoamines, Norepinephrine, Dopamine, Serotonin

## Abstract

Alzheimer’s disease (AD) is a devastating neurodegenerative disorder characterized by relentless cognitive decline and the emergence of profoundly disruptive neuropsychiatric symptoms. As the disease progresses, it unveils a formidable array of neuropsychiatric manifestations, including debilitating depression, anxiety, agitation, and distressing episodes of psychosis. The intricate web of the monoaminergic system, governed by serotonin, dopamine, and norepinephrine, significantly influences our mood, cognition, and behavior. Emerging evidence suggests that dysregulation and degeneration of this system occur early in AD, leading to notable alterations in these critical neurotransmitters’ levels, metabolism, and receptor function. However, how the degeneration of monoaminergic neurons and subsequent compensatory changes contribute to the presentation of neuropsychiatric symptoms observed in Alzheimer’s disease remains elusive. This review synthesizes current findings on monoamine alterations in AD and explores how these changes contribute to the neuropsychiatric symptomatology of the disease. By elucidating the biological underpinnings of AD-related psychiatric symptoms, we aim to underscore the complexity and inform innovative approaches for treating neuropsychiatric symptoms in AD.

**Graphical Abstract [created With Biorender.com]:**

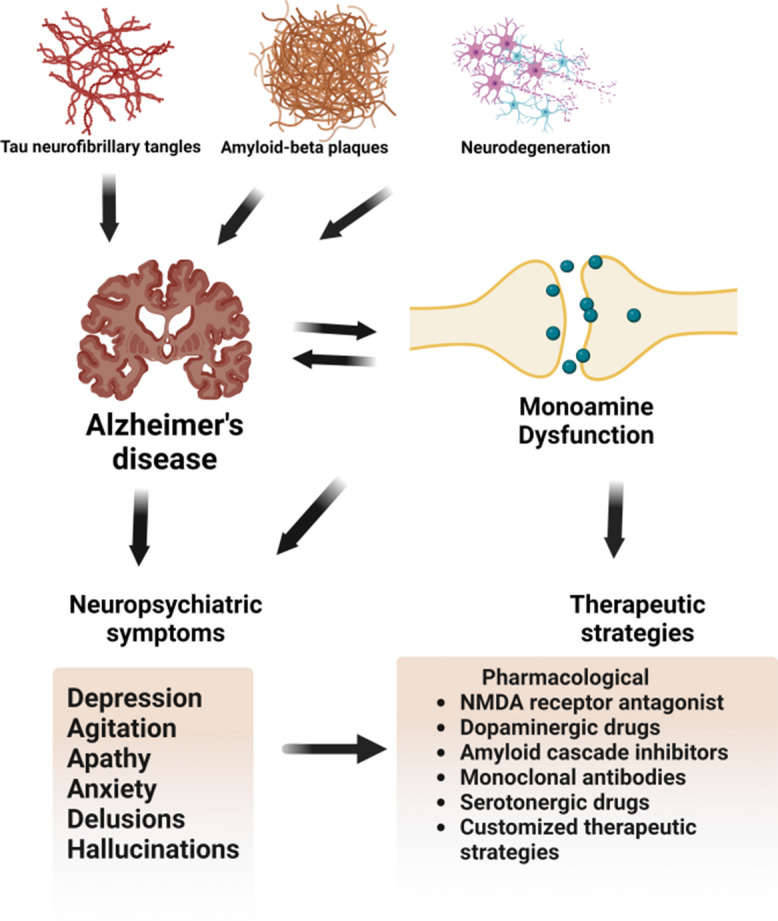

## Introduction

Alzheimer’s disease (AD) poses a growing concern in public health and stands as the most prevalent neurodegenerative condition in aging individuals. The Alzheimer’s Association reports that over 6.9 million Americans currently have AD, with projections indicating an increase to 11–16 million by 2050 [1]. Treating AD comes at a significantly high cost, and inadequate therapeutic approaches can result in considerable harm to both individual health and the economy. Providing care for AD patients imposes a burden three times greater than other elderly disorders [2]. It represents the most common form of dementia, marked by a gradual decline in cognitive function across various domains such as language, attention, and visuospatial cognition [3, 4]. The Food and Drug Administration (FDA) has approved treatments for AD, including cholinesterase inhibitors such as donepezil, rivastigmine, and galantamine, as well as newer drugs like lecanemab and aducanumab. These treatments help manage symptoms but are not curative. The FDA has only approved memantine for moderate to severe stages, which reduces symptoms by regulating NMDA-type glutamate receptors in the brain. [5–7]. Unfortunately, these drugs offer only marginal benefits, merely alleviating symptoms for a while [8].

In addition to cognitive impairment, individuals with AD often experience neuropsychiatric symptoms (NPS), including hallucinations, delusions, apathy, and depression. These symptoms are widespread throughout the spectrum of AD, differing in their progression. The most commonly reported NPS in Alzheimer’s patients is apathy (69.8%), agitation (55.8%), and irritability (48.8%) [4, 9, 10]. Apathy and agitation, measured by the neuropsychiatric inventory (NPI) 3.2 scale, are considered the most severe NPSs. The presence of NPS in AD can exacerbate symptoms, leading to increased disability and burden, potentially contributing to faster progression to severe dementia and even death [11]. In May 2023, the FDA approved brexpiprazole, an atypical antipsychotic, for treating agitation in AD. This approval is significant as it addresses a common and distressing symptom that heavily impacts both patients and caregivers [4]. Despite this progress, there are still no FDA-approved medications specifically for other NPS in Alzheimer’s. As a result, psychotropic medications are often prescribed off-label. The lack of approved treatments is partly due to the limited understanding of the causes of these symptoms and their connection to the underlying brain disease in Alzheimer’s. The neuropathological features associated with AD encompass various types of lesions, including amyloid plaques, cerebral amyloid angiopathy, neurofibrillary tangles (NFT), and neuroinflammatory responses. Additionally, there is a significant presence of neuronal and synaptic dysfunction, primarily affecting the temporal cortex and hippocampus [12]. Any of these factors may alter brain function, and their combined effect ultimately leads to neurodegeneration.

Among the first affected brain regions in AD are monoaminergic systems (MA-ergic), recognized as pivotal cellular targets relevant to various neuropsychiatric and neurological pathologies. The impact of AD on MA-ergic systems has been a longstanding area of interest, with researchers highlighting significant reductions in nucleolar volume and total RNA levels within serotonergic (5-HT-ergic) and norepinephrinergic (NE-ergic) neurons in the brainstem of AD patients [13–15]. Disruptions in functional connectivity, particularly within the default mode network, are associated with monoaminergic signaling alterations involving 5-HT, dopamine (DA), and NE. These disruptions contribute to common neuropsychiatric symptoms such as depression, anxiety, and behavioral changes observed in Alzheimer’s patients [16]. Understanding the causative steps/effects between deficits in monoaminergic systems and changes in functional connectivity is crucial for comprehending cognitive decline, affecting memory, attention, and executive function. Additionally, the identification of polymorphisms in MA-ergic-related genes is associated with behavioral and cognitive features of AD, bringing new dimensions to understanding the disease [13, 17, 18].

Our current knowledge regarding the neurochemical foundation of NPS in AD remains limited. Although cognitive changes and especially early memory deficits frequently trigger neuropsychotic symptoms, like mood changes or agitation, there is growing evidence that the monoaminergic neurons undergo degeneration at the beginning phase of AD [19]. Clinical findings further support this, showing reduced levels of DA, serotonin (5-HT), and norepinephrine (NE), along with their metabolites, in the central nervous system (CNS) [20–22]. These observations underscore the need for a closer examination of the monoaminergic system’s role in AD pathology. These facts merit a closer examination of the role of the monoaminergic system in AD pathology. This article investigates the present corpus of literature addressing the intricate relationship between pathological presentations and NPS in AD, with a primary focus on alterations within the monoaminergic system and how these changes contribute to the manifestation of NPS in individuals with AD. Particularly, the article also examines the intricate compensatory mechanisms linked to the alterations in the monoaminergic system. By uncovering the biological foundations of AD-related psychiatric symptoms, our goal is to highlight the complexity of and inspire novel strategies for treating neuropsychiatric symptoms in AD. Given their well-established roles in mood and affective disorders, this article will primarily focus on NE, 5-HT, and DA. The role of the histaminergic system in AD is also emerging, and its therapeutic potential has been reviewed recently (PMID: 37,123,051). We will not cover histamine in this article.

## NPS and the associated pathological presentations in AD patients

**Fig. 1:**
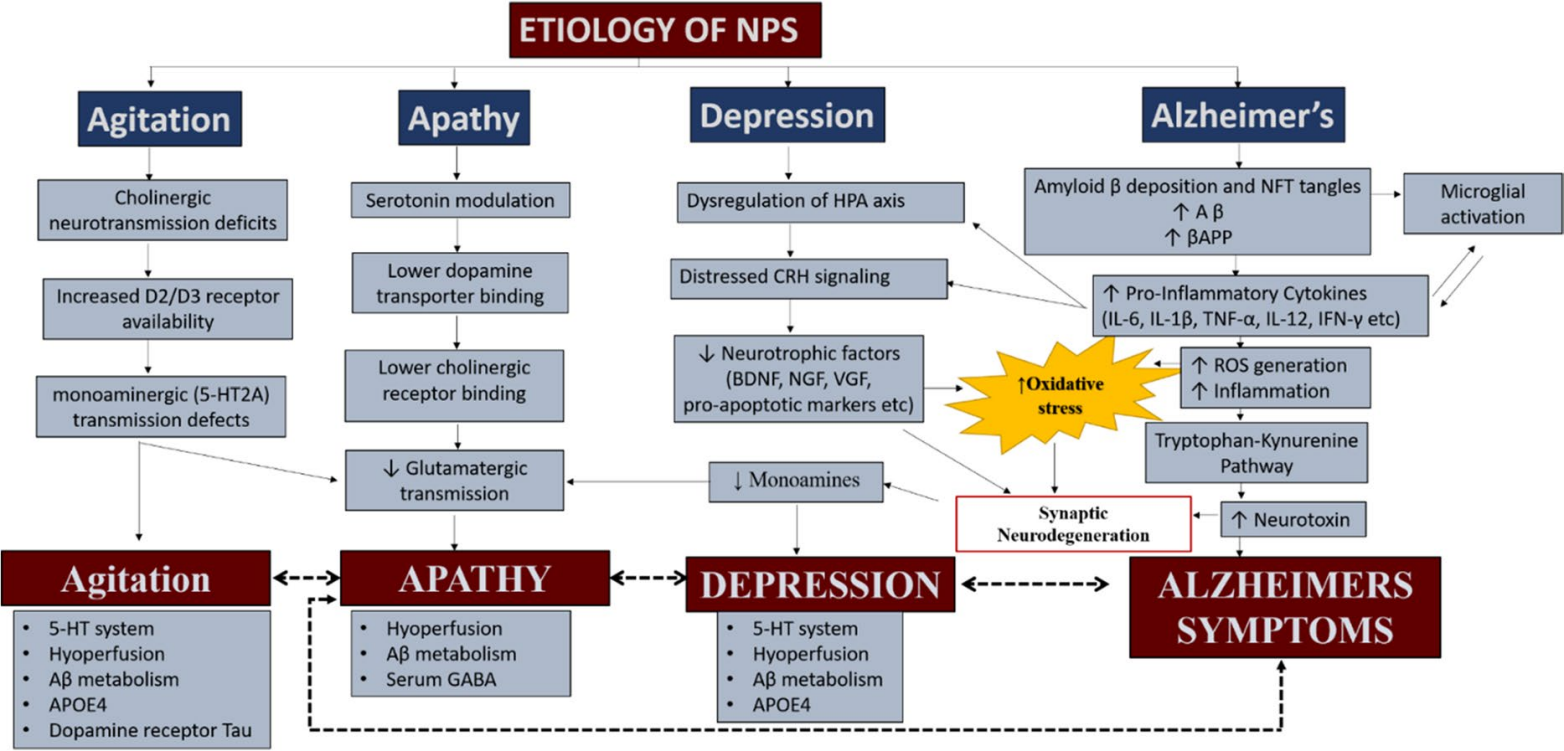
This figure depicts the etiology of neuropsychiatric symptoms in conditions such as Alzheimer’s disease, focusing on three main categories: agitation, apathy, and depression. It illustrates how disruptions in neurotransmitters like acetylcholine, dopamine, and serotonin, along with the accumulation of amyloid-beta and tau proteins, contribute to these symptoms. The diagram highlights key neurochemical changes for each category, including receptor binding alterations, neurotransmitter modulation, and inflammatory responses. Oxidative stress is shown as a central mechanism driving synaptic neurodegeneration. Arrows depict the relationships between various processes, while dotted lines suggest potential interactions among symptom categories. At the bottom of the figure, specific systems and biomarkers associated with each symptom are listed, underscoring the complex and interconnected nature of NPS etiology in neurodegenerative disorders

NPS is linked to the progression of dementia, appearing in three phases: (1) irritability, depression, and nighttime behavior changes; (2) appetite changes, agitation, and apathy; and finally, (3) motor disturbances, hallucinations, and disinhibition [23, 24]. Numerous research studies have examined the neurobiological and neuropathological changes associated with specific NPS in AD (as shown in Table 1) and unveiled consistent patterns across various symptoms (Fig. 1). While the accumulation of amyloid beta plaques, hyperphosphorylation of tau proteins, and the proliferation of NFT are standout neuropathological changes, individual neuropsychiatric symptoms have been linked to distinct brain alterations that are not typical of a healthy aging brain [16, 25]. In the following sections, we summarize the current knowledge about each main NPS symptom and the relevant associated molecular and pathological changes in the brain of AD patients.

**Table 1:** Alterations in the monoaminergic systems in AD pathology: biochemical and NPS changes

Category	Noradrenergic (NE)	Serotonergic (serotonin)	Dopaminergic (DOPA)
Anatomical region involved	- Locus coeruleus (pons) - Prefrontal cortex (frontal lobe) - Hippocampus (medial temporal lobe)	- Raphe nuclei (brain stem) - Hippocampus (medial temporal lobe) - Prefrontal cortex (frontal lobe)	- Substantia nigra (midbrain) - Striatum (basal ganglia) - Frontal cortex (frontal lobe)
Function	- Attention - Arousal - Stress response - Memory	- Mood regulation - Sleep - Appetite - Memory - Sensory perception - Sexual and feeding behavior	- Reward - Motivation - Motor control - Executive function
Biochemical changes	- Decreased NE levels in cerebrospinal fluid - Reduced NE transporter expression - Altered NE receptor density	- Decreased 5-HT levels in cerebrospinal fluid - Reduced 5-HT receptor density - Altered 5-HT transporter expression	- Decreased DOPA levels in the brain - Reduced DOPA transporter activity - Altered DOPA receptor expression
Alteration in NPS	- Depression - Anxiety - Cognitive impairment - Psychosis - Agitation - Insomnia	- Mood swings - Aggression - Sleep disturbances - Agitation - Insomnia - Depression	- Apathy - Motor disturbances (e.g., tremors) - Cognitive decline - Akinesia - Agitation - Rigidity

This table summarizes the biochemical alterations and corresponding NPS associated with dysfunction in the monoaminergic systems in AD pathology, including changes in the norepinephrine (NE), serotonergic (serotonin), and dopamine (DOPA) systems

### Depression

**Fig. 2:**
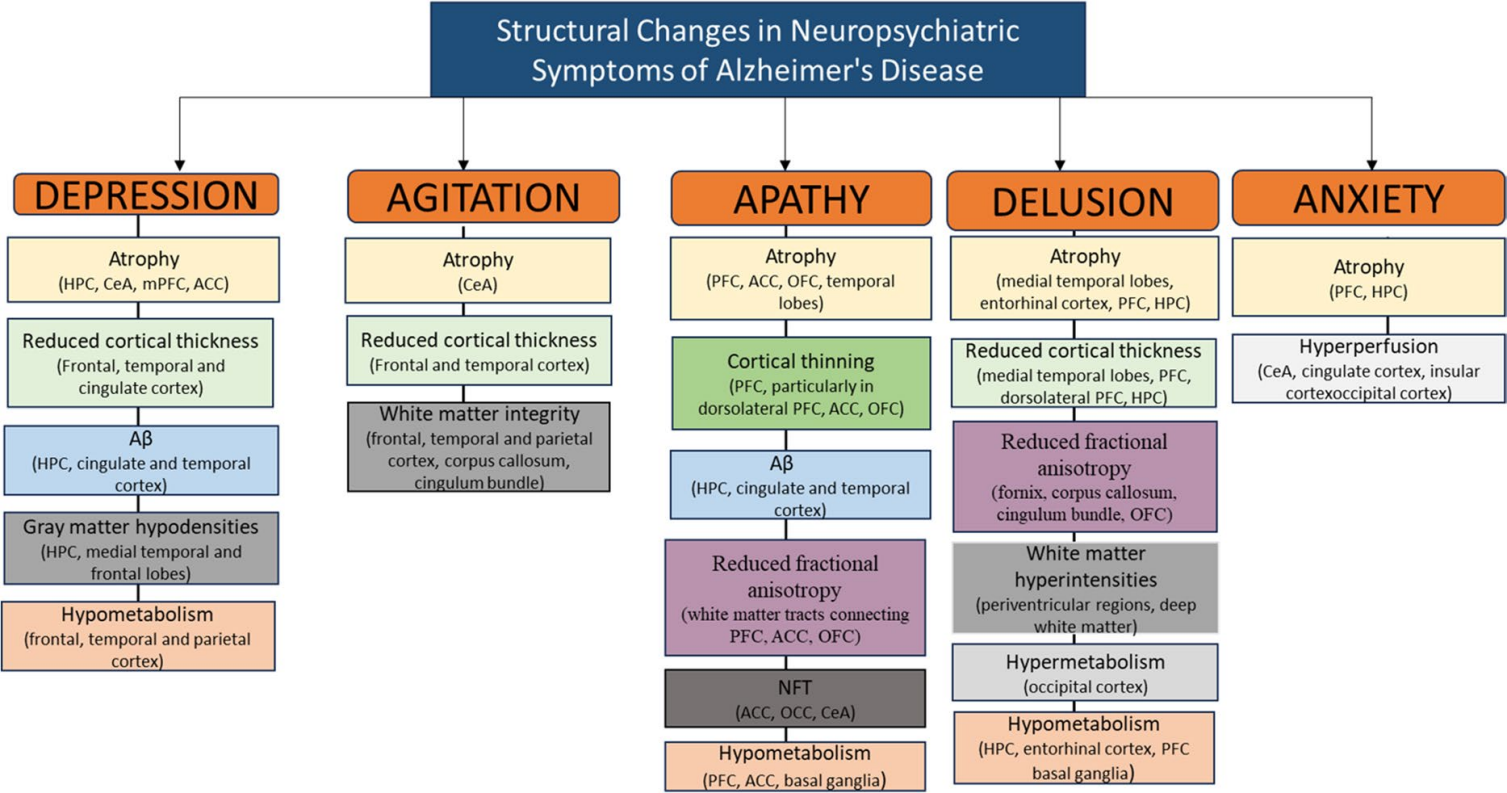
This figure illustrates the structural abnormalities associated with neuropsychiatric symptoms in Alzheimer’s disease, as revealed by neuroimaging. These abnormalities include hippocampal atrophy and cortical thinning, particularly in the medial temporal lobes and prefrontal cortex, which signal neurodegeneration and contribute to the cognitive decline and behavioral changes observed in Alzheimer’s disease patients

Depression is a serious condition affecting millions worldwide, leading to persistent sadness, loss of interest, fatigue, and changes in sleep and appetite. It can worsen existing health problems and increase disability [26]. Depression often co-occurs with AD, with prevalence rates ranging from 25 to 74.9% [16]. Patients with depression exhibited the most pronounced neurobiological changes upon autopsy examination, and those with both depression and AD showed increased plaques, tangles, and a general decrease in cerebral glucose metabolism [16]. Depression in AD could be a psychological reaction to AD or stem from the same disease processes causing other symptoms (such as abnormal beta-amyloid (Aβ) processing, tau hyperphosphorylation, etc.). Furthermore, these patients presented with hippocampal sclerosis, diminished GABA levels, and reduced cortical thickness of the EC [16, 27]. Depression in AD is linked to accelerated atrophy in the anterior cingulate cortex (ACC) and reduced 5HT1-A receptors [25]. This can cause gray matter loss in the frontal and temporal lobes, impacting areas like the prefrontal and cingulate cortexes, and inferior temporal gyrus [25]. Depression also leads to white matter lesions in the frontal, parietal, and temporal lobes, along with hypometabolism in certain regions (Fig. 2) [28, 29]. Research often associates depression in AD with frontal-striatal and subcortical limbic circuits, involving dorsal, ventral, and rostral ACC [30, 31]. Correlation analysis of the brain with single photon emission computed tomography (SPECT) and the NPI score revealed a connection between depression in AD and a spot in the left frontal gyrus [32]. Some studies suggest that the hippocampus undergoes structural changes in depression and is therefore the primary site responsible for it [33, 34]. This forms a hippocampal-prefrontal cortex model for mood regulation and cognitive dysfunction, emphasizing the role of the limbic system in AD-related depression [33].

### Agitation

Agitation is a behavioral condition that involves restlessness, aggression, and emotional distress. It affects 30–50% of Alzheimer’s patients and is the third most common non-pharmacological symptom after apathy and depression. Currently, there are no safe and effective treatments available [35]. Agitation is a problematic behavior for AD caregivers, yet it is challenging to define and measure. Evidence suggests that agitation/aggression in AD is connected to dysfunction in specific brain regions responsible for emotional regulation, such as the frontal, anterior cingulate, and posterior cingulate cortices, as well as the amygdala and hippocampus [36]. These changes can cause heightened threat perception and emotional dysregulation, leading to hypervigilance. Agitation and aggression in AD are also linked to decreased cholinergic and 5-HT-ergic markers, increased tau proteins, and changes in the N-acetyl aspartate/creatine and myoinositol/creatine ratios [36]. Additionally, there is increasing evidence indicating that deficits in cholinergic neurotransmission, beyond those observed in AD itself, and heightened availability of D2/D3 receptors in the striatum may be linked to structural and functional deficits associated with agitation symptoms [29, 37]. Agitation and aggression in AD were also correlated with 5HT2-A receptor polymorphism [37] and grey matter atrophy in bilateral anterior cingulate, left insula, and right middle frontal gyri [29, 37–39]. Overall, agitation/aggression in AD is linked to cortical dysfunction in key brain areas (Figs. 1 and 2) [29, 40].

### Apathy

The presence of apathy has been observed in AD-diagnosed patients characterized by different degrees of executive function impairment, high levels of cerebrospinal fluid (CSF) tau, and phosphorylated tau with low levels of Aβ1-42 (Fig. 1), and these patients show limited or absent response to pharmacological treatment with cholinesterase inhibitors [41–44]. Very high levels of CSF tau protein and the occurrence of apathy were related to a more rapid progression of cognitive decline [45]. State-of-the-art structural and functional neuroimaging techniques, crucial for delving into the fundamental pathological processes of AD, are now being employed to explore NPS in AD [31].

Recent reviews have extensively delved into imaging techniques, including structural magnetic resonance imaging (sMRI), fluid-attenuated inversion recovery, diffusion tensor imaging, fluorodeoxyglucose positron emission tomography (FDG-PET), Pittsburgh compound B positron emission tomography, carbon-11-labeled PBB3 positron emission tomography, SPECT, resting-state functional magnetic resonance imaging, and task-based functional magnetic resonance imaging, to explore apathy in individuals with AD. These investigations identified the involvement of the frontostriatal circuit, particularly implicating the ACC, prefrontal cortex (PFC), and components of the basal ganglia, such as the ventral striatum (including the nucleus accumbens (NAcc) and olfactory tubercle) [29, 31]. Correlations between apathy and various markers were observed, including hypoperfusion in the ACC, orbitofrontal cortex (OFC), ventromedial PFC, putamen, and posterior cingulate cortex (SPECT and FDG-PET); cortical thinning and lower volume in these regions (sMRI); and lower fractional anisotropy in the corpus callosum, longitudinal fasciculus, and uncinate fasciculus (diffusion magnetic resonance imaging) (Fig. 2). Apathy also exhibited associations with increased Aβ depositions in the ACC, PFC, and putamen, as well as heightened tau accumulation in the OFC [29, 31]. Moreover, the severity of apathy was linked to gray matter atrophy in both the bilateral ACC and left medial frontal cortex [46]. Diminished gray matter density was evident in the bilateral ACC, frontal cortex, left caudate nucleus, and bilateral putamen [39]. A decrease in cortical thickness, correlating with apathy severity, was observed in the left caudal ACC, left OFC, left superior and ventrolateral frontal regions, as well as the inferior temporal region [31]. Alzheimer’s patients with apathy exhibited lower DA transport binding, an overall decrease in cholinergic receptor binding in the frontal cortex, reduced metabolic activity in the ACC, and greater white matter volume [29, 47].

### Delusion and hallucinations

NPS within AD are notably dominated by profound elements like delusions and hallucinations. Strikingly, in the initial stages of AD, these intense manifestations tend to be less commonplace [47]. Bruen et al. showed that certain NPS in early AD are linked to brain structure atrophy [39]. Symptoms like delusions and agitation are more common in later stages but can also appear in mild AD [39]. In sync with the correlation found with aggression, psychosis is associated with variations in the 5-HT system. Individuals experiencing hallucinations and delusions, for instance, show an elevated prevalence of the C allele and CC genotype of the T102C variant of 5HT (2A) receptors [48]. Conversely, patients reporting symptoms of psychosis exhibited not only increased tangles, plaques, and Lewy body disease but also an elevated density of DA-3 receptors and heightened hyperphosphorylation of tau proteins [23]. Using SPECT, Banno et al. [49] found psychosis symptoms in AD were associated with reduced cerebral blood flow in the right angular gyrus and right occipital lobe. Similarly, other studies have found significant associations between regional atrophy and psychosis onset, covering delusions, agitation, wandering, hallucinations, and the need for anti-psychotic medications (Fig. 2) [50].

### Anxiety

Anxiety is an early symptom of AD, a risk factor for dementia, and its neurobiological origin in AD is uncertain. Some studies suggest that AD neuropathology might be the root cause rather than anxiety-induced hippocampal damage due to elevated cortisol levels [51]. While MRI investigations have not consistently detected changes in gray matter associated with anxiety in AD [52], they have indicated the presence of white matter hyperintensities, pointing to a possible vascular component [53, 54]. Certain studies indicate a connection between anxiety and a decrease in the volume of the entorhinal cortex (EC) [51]. PET imaging has revealed that heightened anxiety is linked to reduced metabolism in specific brain regions, such as the EC, anterior para-hippocampal gyrus, superior temporal gyrus, and insula [54]. Taken together, it appears that anxiety in AD may be related to the medial temporal lobe, with a particular focus on the entorhinal region.

The comprehensive body of research encapsulated in Table 1 provides a detailed overview of the associations between specific neuropsychiatric symptoms and the observed neurobiological and neuropathological changes in AD. An intriguing observation arises from the data focused on agitation, depression, apathy, and psychosis in AD—a convergence of regions (e.g., frontal cortex, cingulate cortex, caudate nucleus, amygdala) linked to each NPS syndrome. Notably, these regions have common associations with the salience network (anterior cingulate and insula), mood regulation (amygdala), and motivated behavior (frontal cortex). These commonalities suggest an overlap in the brain mechanisms underlying these diverse NPS. Moreover, these neuropsychiatric symptoms are complexly tied to the malfunction of monoaminergic system pathways relevant to AD, including the DA system, 5-HT system, and NE system. Next, we delve deeper into the monoaminergic alterations associated with AD progression, unveiling a narrative that weaves together the complexities of neurobiology, neuropathology, and the intricate web of neuropsychiatric manifestations.

## Alterations of MA-ergic systems and their implications for NPS in AD

In AD, MA-ergic neurons are highly vulnerable and degenerate in the early stages of the disease. Their long, poorly myelinated axons increase the risk of developing NFT [55]. When DA and NE oxidize, they create neuromelanin, which can exacerbate AD pathology and oxidative stress [56, 57]. Over time, neuromelanin accumulates and gets released by dying neurons, which can contribute to neurodegeneration by releasing chelated molecules into the extracellular space, thereby triggering inflammation [56, 57]. Additionally, the interaction among diverse MA-ergic systems involves shared input and output regions, establishing direct projections [55]. The interplay among these systems, specific to both brain regions and receptors, suggests that certain features of NPS in AD may be connected to network changes controlled by monoaminergic neurotransmitters (Table 1). In the sections that follow, we explore each monoaminergic system in detail, synthesizing current research on alterations in monoamines in AD and examining how these changes contribute to the neuropsychiatric symptoms associated with the disease.

### Alterations in the noradrenergic system in AD

**Fig. 3:**
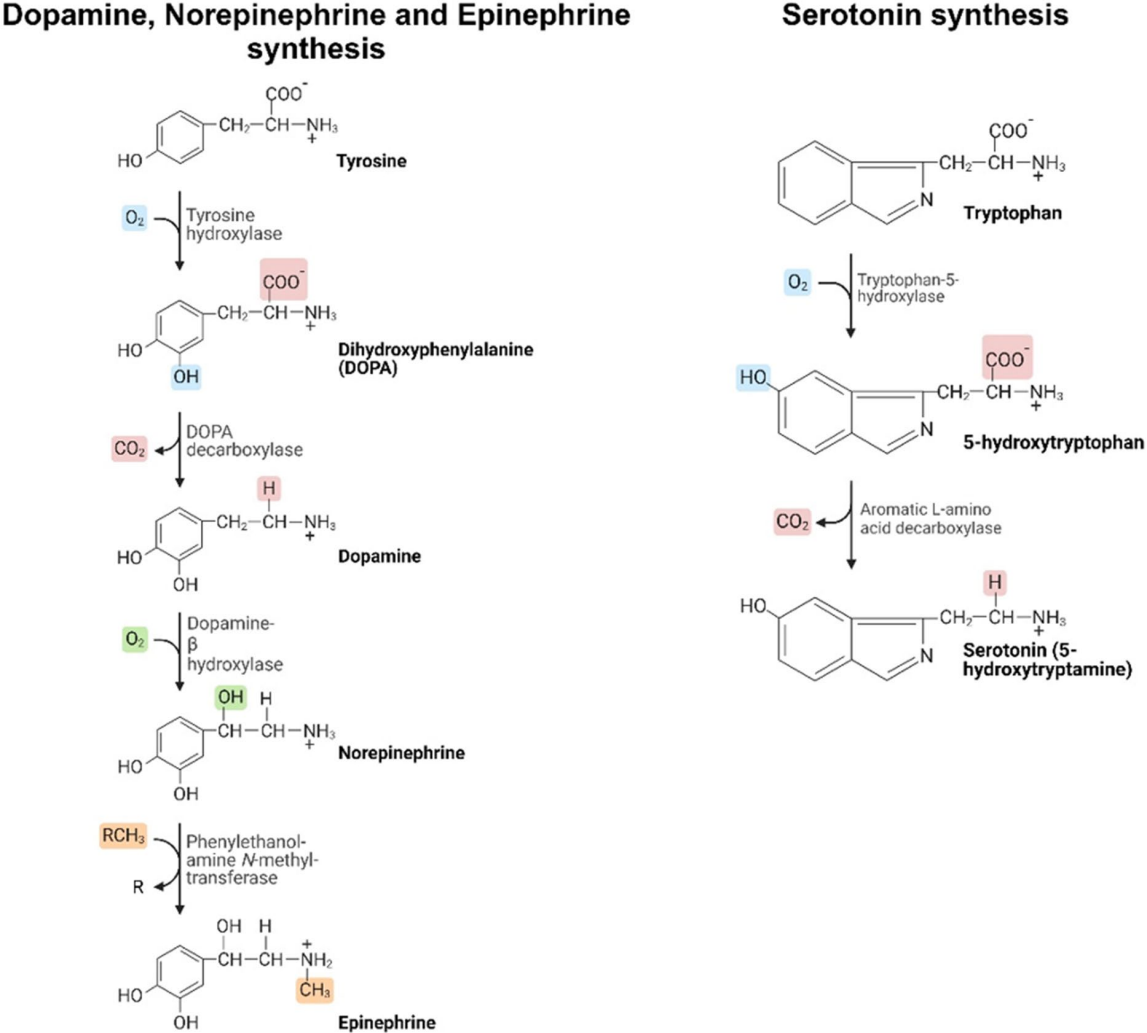
Illustration of neurotransmitter biosynthesis. Dopamine is produced from tyrosine-by-tyrosine hydroxylase and dopamine decarboxylase, while serotonin is synthesized from tryptophan-by-tryptophan hydroxylase and aromatic amino acid decarboxylase. Disruptions in these synthesis pathways can affect brain function and contribute to symptoms of neurodegenerative diseases such as Alzheimer’s. [Created with Biorender.com]

In the brain, the noradrenergic system plays a crucial role in regulating normal brain functions, primarily originating from locus coeruleus (LC) and exerting widespread influence over various brain regions. NE, derived from the amino acid tyrosine through a series of enzymatic reactions (see Fig. 3), is a monoamine frequently linked to depression and other mood disorders in AD [58]. Positioned beneath the fourth ventricle in the dorsal pons, the LC is particularly susceptible to damage if exposed to harmful substances and neuroinflammatory molecules [59]. The axons of LC neurons are thin, extensively branched, and unmyelinated, and are among the longest in the brain, facilitating widespread innervation of the entire cerebral cortex and exerting influence over various brain regions [60]. Alongside the nucleus basalis of Meynert (NBM), neocortex, EC, and hippocampus, the LC’s vulnerability potentially contributes to early impairments in declarative memory [61, 62].

In patients with AD and mild cognitive impairment (MCI), the first signs of involvement of the LC were detected through atrophy and a reduction in volume [63]. This reduction was associated with the subsequent development of AD within 2 years [63]. In terms of spatial distribution, AD affects the caudal region within the LC. Studies have shown that the rostrocaudal extent of the nucleus shrinks by an average of 6 mm in AD patients [64, 65]. MRI contrast of the LC was found to be reduced in AD, correlating with levels of CSF amyloid beta [66].

The participation of the LC-NA system is apparent in various cognitive and psychiatric symptoms observed in neurodegenerative diseases. These disruptions in noradrenergic activity followed phases of heightened activity and slowed dynamics [64]. A significant instance is the simultaneous occurrence of apathy and impulsivity, both associated with the LC-NA system in neurodegenerative disorders like Alzheimer’s, Parkinson’s, and progressive supranuclear palsy [64, 67].

Changes in NE levels are associated with NPS in AD. Drugs that target the NE system often show effectiveness in managing symptoms such as agitation, aggression, and depression. Direct evidence linking NE with depression was found in genetic studies with NE transporter (NET) knockout mice and human polymorphism studies [68–70]. A study by Solich et al. [71] identified differentially regulated gene groups in the brains of NET-knockout mice compared to wild-type mice. According to RT-PCR studies, expression of Fos and Nr4a1 was elevated in response to antidepressants [71]. Furthermore, inhibiting the NE transporter pharmacologically was shown to increase the expression of these genes [71]. Additionally, other genes such as Adra2a, Adra1d, Htr2c, Gria1, Gria3, Scn2a1, Ntrk2, Caly, Ndrg2, Crhbp, Sst, Penk1, Npy, Ntf3, and Egr1 also exhibited altered expressions in NET-KO mice [71]. The Adra1d and Adra2a genes, which encode adrenergic receptors, were upregulated, consistent with previous findings [72].

The presence of both positive and negative symptoms, such as apathy, impulsivity, agitation/anxiety, and depression, indicates an imbalance in the regulation of engagement and disengagement with the environment. Hypoactivity in the LC limits the exploration of new stimuli due to insufficient sustained activity. This hypoactivity can result from cell loss, decreased release capacity of NE, or increased inhibition [64]. This may explain why apathy can manifest at different stages of the disease progression [64, 73]. While apathy is a common feature across age-related neuropsychiatric disorders, it can also occur in the earliest and even presymptomatic stages [74]. Conversely, hyperactivity in the LC may contribute to an anxious or agitated phenotype. The LC-NA system responds to stress by increasing sustained activity, prompting the organism to engage in sampling and scanning behaviors [64]. Elevated baseline activity of this system alone can induce anxiety-like behaviors, as shown in animal studies [75]. Moreover, the NA system exhibits a strong connection to aspects of impulsivity related to restraining or halting ongoing actions [64, 76]. Within this framework, the significance of noradrenaline in restructuring extensive networks becomes especially apparent, given that the involvement of prefrontal-striatal networks is essential for effective inhibition [64]. Additionally, besides being a neurotransmitter, NE is also known to be an anti-inflammatory agent within the CNS and, when depleted, can exacerbate the Aβ-induced neuroinflammation [77].

At the molecular level, a significant loss of noradrenergic neurons in the LC was documented in AD, correlating with cognitive decline, as evidenced by mRNA expression for tyrosine hydroxylase (TH), α2A-AR, and NET, along with morphometric analysis [65, 78–81]. Recent technological advancements, like high-resolution fast spin-echo T1-weighted imaging, not only detect decreased density in the LC but also enable earlier AD detection, supporting evidence of early LC degeneration [82, 83]. LC vulnerability initiates neurodegenerative processes, diminishing NE levels, exacerbating neuroinflammation, impairing amyloid beta clearance, and worsening cognitive decline in AD [82]. Symptom severity correlates with LC neuronal loss, highlighting LC-NA pathway dysfunction [66, 82–84].

One study found a decrease in NE transporters, preventing norepinephrine from re-entering presynaptic neurons, potentially explaining the observed increase of norepinephrine in the synapse [85, 86]. These contrasting findings highlight the need for further research on this topic. Some authors have shown an increase in neuritic plaque density and dysregulation of structural plasticity in the LC of post-mortem AD tissue samples with increasing disease progression [87]. Recent data have demonstrated that LC degeneration causes a retraction of microglia processes both in their resting and activated states via impaired signaling at α2- and β2-adrenoceptors [88, 89]. The degenerative loss of this neurotransmitter in the CNS leads to microglial dysfunction and increased neuroinflammation [90].

Human studies have demonstrated a topographic correlation between LC neuronal loss along the rostro-caudal axis and the distribution of Aβ plaques in the cerebral cortex of AD brains [90]. Post-mortem analyses further reveal that the loss of LC neurons in specific regions corresponds to an increased Aβ plaque burden in the frontal, temporal, and occipital cortices [90]. Despite LC degeneration, compensatory physiological and metabolic responses, such as increased dendritic sprouting of NE neurons to target regions, elevated levels of NE-synthesizing enzymes like TH and dopamine beta-hydroxylase, and reduced levels of NET, are thought to significantly negate the NE levels drop from LC deterioration [90, 91]. These compensatory mechanisms can not only delay sudden NE level drops in AD patients but can often overcompensate and cause NE levels to rise, especially in the cerebrospinal fluid [91–93]. Elevated NE level in cerebrospinal fluid is directly proportional to AD severity and cognitive decline [91–93]. These seemingly contradictory roles in different AD NPS suggest that NE has an important role in AD pathogenesis.

The NA system is not only a well-recognized sensitive target of Aβ and tau toxicity; Zhang et al. have provided compelling insights into the direct etiological role of noradrenergic signaling in AD pathogenesis [94]. It has been demonstrated that AβO redirects NE-elicited signaling via α_2A_AR to trigger the pathogenic GSK3β/tau cascade, resulting in tau hyperphosphorylation and hastened cognitive decline [94]. This mechanism allows AβO to activate GSK3β even at low concentrations, potentially initiating AD pathology in its early stages [94]. Pharmacological blocking of NE/α_2A_AR signaling effectively reduces GSK3β/tau cascade activation and alleviates cognitive deficits, suggesting α_2A_AR blockers as promising treatments for AD [94]. Targeting the Aβ-α_2A_AR interaction offers a disease-specific therapeutic avenue, with combined α_2A_AR blockers and Aβ-reducing drugs showing potential synergy [94]. In addition to regulating Aβ proteotoxicity, activation of α_2A_AR promotes Aβ generation through disrupting APP and sortilin-related receptors with A-type repeats (SORLA) interaction [95]. These discoveries shed light on NE involvement in AD pathogenesis, holding significant implications for future drug development and trial interpretation.

Under normal circumstances, inhibiting both β1 and β2 adrenergic receptors (βARs) with propranolol can lead to memory and learning difficulties [96–100]. Interestingly, in animal models of AD, blocking βARs appears beneficial. Specifically, activation of β2AR enhances the production of Aβ, whereas blocking β1AR reduces its levels [101, 102]. However, the outcomes of these interventions vary across different AD models [101, 102]. Similarly, α1AR activation has been associated with cognitive enhancement in wild-type animals, but blockade of these receptors with prazosin improves cognitive behaviors in AD transgenic mice (APP23) [103]. In AD patients, α1AR activation exacerbates agitation symptoms, with prazosin demonstrating efficacy in alleviating such symptoms [104].

AD is associated with substantial alterations in adrenergic receptor expression, signaling, and downstream effectors, indicating dysregulated NE receptor function independent of NE levels [91]. Understanding the dynamic changes in LC compensation, NE tone, and adrenergic receptor function is crucial for elucidating noradrenergic contributions to AD pathogenesis. Therapeutic strategies targeting NA signaling or preventing LC degeneration could represent a new paradigm for disease-modifying treatment.

### Alterations in the serotoninergic system

**Fig. 4:**
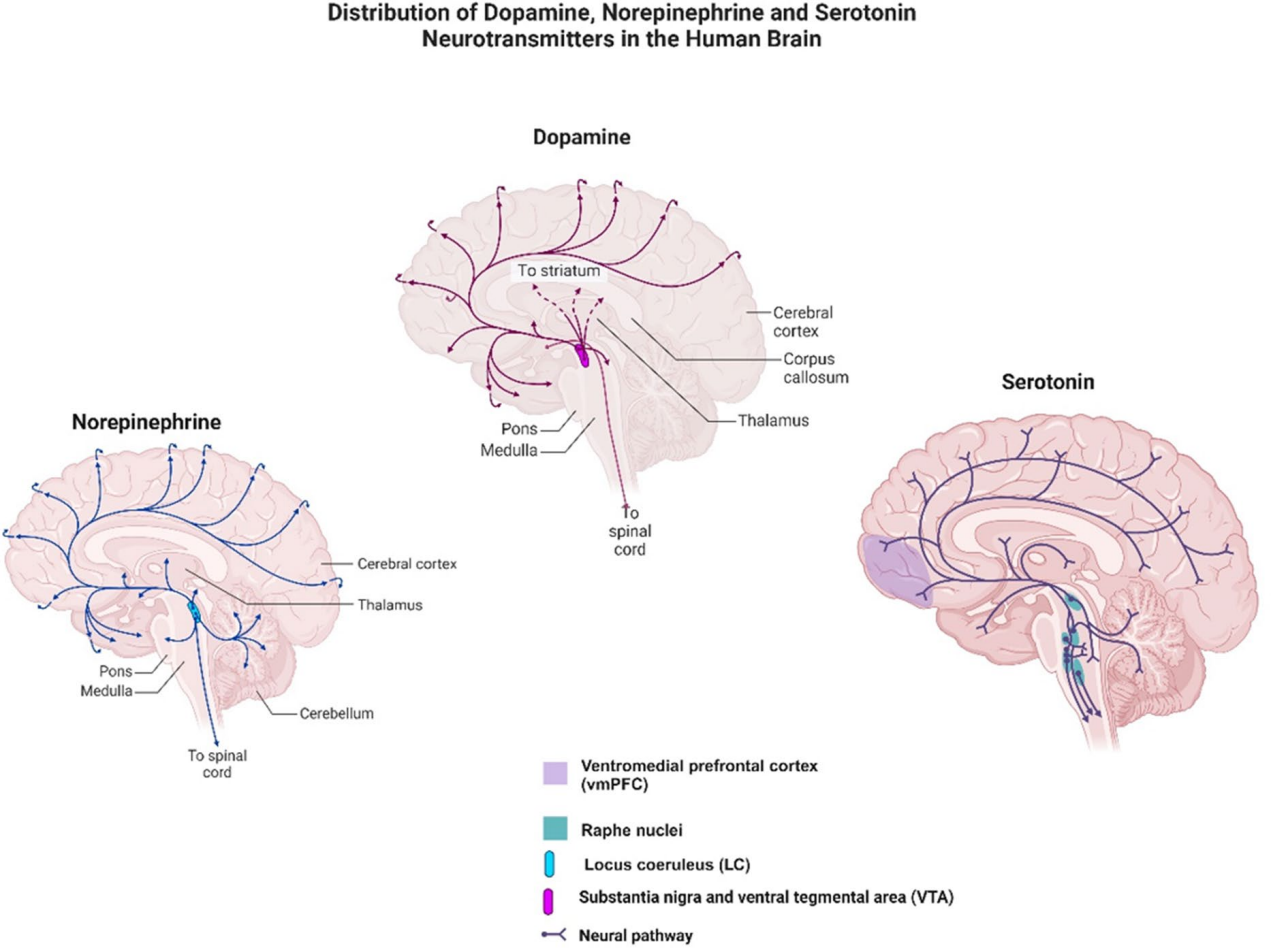
Key components of the monoaminergic circuitry in the brain, emphasizing its crucial role in regulating diverse physiological functions and influencing neuropsychiatric states. The intricate network encompasses serotonin, dopamine, and norepinephrine pathways originating from specific nuclei, including the raphe nuclei, VTA, substantia nigra, and LC. Serotonergic projections impact mood and emotion, while dopaminergic pathways contribute to reward, reinforcement, and motor control. The noradrenergic system, stemming from the LC, modulates arousal, attention, and stress response. [Created with Biorender.com]

Serotonin, also known as 5-hydroxytryptamine, plays a crucial role as a neurotransmitter in various physiological functions, including mood regulation, appetite, and sleep. It is primarily synthesized in the raphe nuclei located in the brainstem. The projections from the raphe nuclei extend to several brain regions, including the cerebral cortex, thalamus, hypothalamus, and basal ganglia, as well as descending to the brainstem and spinal cord [105] (Fig. 4). These projections are involved in regulating mood, attention, and other cognitive processes. Specifically, serotonin projections from the median raphe primarily target midline forebrain regions, such as the dorsal diagonal band of Broca nuclei, medial septum, and dorsal hippocampus, playing a regulatory role in anxiety, fear, and memory within structures like the amygdala, hippocampus, and septum [105, 106]. Additionally, serotonin neurons extend to areas such as the limbic system, spinal cord, and autonomic nervous system, influencing various physiological functions, including pain modulation, motor function, blood pressure regulation, heart rate, and digestion [105, 106].

Perturbations in 5-HT-ergic systems are associated with memory decline in AD, impacting cognition through their activity in the hippocampus and prefrontal cortex [105]. Apathy, commonly linked to serotonergic degeneration, may manifest independently or in conjunction with depression and anxiety, both of which are key components of 5-HT-ergic system dysfunctions [105]. Yohn et al. suggested the involvement of various 5-HTRs in depressive symptoms, evident in both human and animal models [107]. The impact of SSRIs on apathy-related motivational deficits, distinct from anhedonia in depression, remains controversial. Furthermore, evidence indicates a correlation between anxiety disorders, such as obsessive–compulsive disorder, and HTR3 variants [108]. Additionally, overexpression, increased density, and mRNA content of NAc 5-HTR4 have been associated with triggering anorexic behaviors [105]. Recent clinical studies indicate reduced intracerebral 5-HT release in depressed patients [109]. Enhancing 5-HT function with drugs like 5-HT receptor agonists and reuptake inhibitors has been proven effective in alleviating depressive symptoms [110]. Initiating SSRIs early, ideally, before plaque deposition, has been shown to reduce the risk of AD in human studies [111–113]. However, comprehensive trials are needed to optimize SSRI doses and duration for influencing disease onset and biomarkers. Notably, the anticholinergic nature of paroxetine raises concerns about cognitive effects in the elderly, warranting further exploration. The properties of SSRIs, particularly 5-HT-ergic vs. anticholinergic, need careful consideration in future trials. Newly developed antidepressants like vortioxetine offer promising avenues for understanding and treating AD [111].

Positive syndromes like agitation, irritability, aggression, disinhibition, and impulsivity are also associated with extensive 5-HT-ergic deficits, and deficiency of serotonin in certain brain areas has been implicated in hyperactivity. Reductions in both 5-HT and its metabolite 5-hydroxyindoleacetic acid (5-HIAA) are associated with overactivity in Brodmann’s area 10 of AD patients [105]. A few studies have reported that 5-HTR2C is associated with delusions and hallucinations in AD patients [114, 115]. Serotonin functions by interacting with cell membrane-bound 5-HT receptors, a mechanism present in human nerve cells [116]. These receptors are widespread throughout the brain, including regions impacted by AD [116]. The prefrontal cortex and hippocampus, the primary targets of 5-HTergic neurons, express nearly all classes of 5-HT receptors (5-HT1–5-HT7) [117].

The signaling of serotonin in AD involves complex presynaptic and postsynaptic mechanisms. While the exact mechanisms underlying alterations in serotonin signaling in AD are not fully understood, a growing body of research has shed light on some of the key changes that occur. Presynaptically, there is a decrease in the density of serotonin terminals and the number of serotonin neurons in various brain regions (dorsal and median raphe nuclei), leading to reduced serotonin release and signaling throughout the brain, contributing to the cognitive and behavioral symptoms seen in AD [118–122]. Alterations in the 5-HT1A receptor subtype, particularly relevant to Behavioral and Psychological Symptoms of Dementia (BPSD) in AD, emphasize its complex role in AD pathology [118]. Studies report changes in the functioning of monoaminergic neurotransmitters and brain metabolism underlying depression in AD. For example, Lai et al. reported selective loss of 5HT1A receptors in the hippocampus, along with loss of 5-HT-ergic and noradrenergic neurons in the raphe nucleus and LC [120, 123, 124]. Early AD may exhibit functional loss of 5-HT-ergic neurotransmission affecting 5HT2A receptors [119, 125]. Interestingly, 5HT2A receptors are indicated in the positive effects of psychedelic therapies on NPS, like those using psilocybin [126]. Some studies suggest a correlation between lower 5-HTT transporter density and greater depression symptom severity. These findings contrast with late-life major depression, suggesting decreased monoaminergic neurotransmitter function and fronto-parietal metabolism in AD-related depression, opposite to late-life depression [127, 128].

In AD with psychosis, reduced 5-HT levels and decreased adenylate cyclase activity after stimulation of 5-HT6 receptors in the ventral temporal cortex and pro-subiculum have been reported [129–131]. The reduced levels of 5-HT could be correlated to lower cell counts in the dorsal raphe nucleus [129]. Additionally, it has been associated with an increased ratio of AChE/5-HT.

Postsynaptically, there is evidence suggesting that alterations in serotonin receptor signaling may also contribute to the pathophysiology of AD. The 5-HT3 receptor-expressing neurons found postsynaptically are mainly GABA cells in the neocortex, olfactory cortex, hippocampus, and amygdala [132] and influence anxiety and psychosis, yet no loss was demonstrated in AD patients’ amygdala and hippocampus compared to controls [133]. The 5-HT4 receptor, implicated in learning and emotion, has not shown alterations in AD [134]. The functions of 5-HT5, 5-HT6, and 5-HT7, along with their role in AD, remain unclear. Receptor-specific ligands for 5-HT5, 6, and 7 are currently in development [116, 118, 135]. In individuals with MCI and AD, a progressive decline in 5-HTR1A density occurs in the hippocampus and dorsal raphe [136]. Post-mortem analysis of AD patient brains reveals reduced 5-HTR1B density in the frontal and temporal cortex, linked to cognitive dysfunction [137]. The observed lower expression of 5-HTR1B in AD may represent a compensatory mechanism to counter downregulated acetylcholine levels since these receptors inhibit acetylcholine release [137].

Studies using immunohistochemical, post-mortem, and imaging approaches show a reduced brain 5-HTR2A density in AD patients correlating with cognitive impairment severity [138–140]. Alterations in 5-HTR2A receptor expression are associated with the age-related accumulation of Aβ plaques, and modulating 5-HTR2A receptors can influence cognitive performance [141]. Activation of 5-HTR2A with the agonist TCB-2 enhanced working memory in rats [142], while the 5-HTR2A/2C antagonist ritanserin improved spatial learning and memory in rats [143]. Additionally, the 5-HTR2A antagonist EMD 281014 improved working memory in both young and old monkeys [144]. Additionally, reduced serotonin receptor density, particularly in the hippocampus, may impair postsynaptic response and contribute to cognitive and behavioral symptoms in AD [116]. Medications targeting serotonin receptors, like SSRIs, have shown promise in improving mood and cognitive function by mitigating serotonin dysregulation caused by amyloid beta plaque deposition [13, 29, 118].

### Alterations in the dopaminergic system

DA neurons, primarily located in the midbrain (A8–A10 areas, including the retrorubral field, substantia nigra pars compacta (SNc), and ventral tegmental area (VTA)), exhibit both anatomical and functional heterogeneity. These neurons project to various brain regions, with VTA neurons primarily connecting to the NAcc, amygdala, and cerebral cortex, while SNc neurons project to the dorsal striatum [145, 146]. The nigrostriatal pathway (Fig. 4), influenced by DA, plays a significant role in motor function control and skill learning [147]. Excitatory inputs to the SNc cells come from the cortex, subthalamic nucleus, and pedunculi-pontine nuclei, while VTA neurons (mesocortical-limbic pathway) receive excitatory inputs from the cortex and the pedunculo-pontine nucleus, while the primary inhibitory inputs come from the NAcc and local interneurons [148]. Pathological changes in the mesocortical-limbic pathway contribute to cognitive and behavioral manifestations in the progression of AD [147]. Meanwhile, the nigrostriatal pathway, strongly influenced by DA, significantly regulates motor function control and skill acquisition [147], and it is the primary damaged pathway in Parkinson’s disease leading to typical motor coordination symptoms. Interestingly, one study has shown the increased NE in the substantia nigra in AD with psychosis [130].

Dysfunction in the DA system can occur at various levels during any phase of AD. Behaviorally, DA intricately modulates the brain’s reward circuitry, exerting profound effects on cognition, motivation, and emotion, and contributing to conditions such as addiction and mood disorders, including depression [52]. A recent study suggests that AD may initiate the age-related reduction of DA-ergic neurons in the VTA, independent of Aβ pathology. The death of these DA neurons decreases DA levels in the hippocampus [147, 149]. Conversely, decreased DA levels in the hippocampus contribute to the characteristic memory loss and cognitive decline observed in AD [150].

The complex network of brain regions associated with NPS in AD, such as the posterior and anterior cingulate cortex, medial orbitofrontal cortex, and striatum, all contain monosynaptic DA-ergic projections from the VTA in the midbrain [150–152]. These DA-modulated regions are heavily involved in apathy and depression, indicating that NPS is partially characterized by hypodopaminergic symptoms [151, 152]. Apathy is a prevalent behavioral abnormality in over 70% of individuals with AD [153], stemming from dysfunction in the DA system, a pivotal component of the brain’s reward system. This underscores the profound impact of DA malfunction on behavior [153]. Apathy often emerges as a secondary effect of DA dysfunction, serving as a notable indicator of disease progression in individuals with mild cognitive impairment and those diagnosed with AD [153, 154]. The onset of these clinical conditions is linked to the accumulation of Aβ burden, and they may coexist within the same individual [153, 154]. While apathy is commonly observed, not all individuals with AD will experience apathy symptoms during their lifetime. Approximately half of AD patients exhibit apathy during the early to mid-stages of the disease [147]. Maintaining an optimal DA level is crucial for the normal operation of DA-ergic pathways, as both excessive and insufficient levels can lead to pathological symptoms.

AD patients with delusions experience significant neurodegeneration in the cingulo-striatal and medial temporo-parietal regions, suggesting a complex neuropathological mechanism potentially linked to DA-ergic dysfunction [155]. Notable tissue loss was found in the mesocorticolimbic pathway, particularly in the ventral striatum, orbitofrontal cortex, and medial temporal lobe, with this atrophy stabilizing at the MCI-AD stage [156]. The degree of atrophy in these mesocorticolimbic regions was linked to higher levels of depression, anxiety, and apathy in both MCI-AD and dementia-related AD patients [156]. Additionally, changes in metabolic connectivity were found between the ventral striatum and fronto-cingulate regions in patients with dementia-related AD but not in those with MCI-AD. No significant changes were observed in the nigrostriatal pathway [156].

DA receptors, which are G protein-coupled receptors, mediate DA’s effects in the central and peripheral nervous systems. Primarily located in the pituitary gland and brain, especially in the basal forebrain, the activation of DA receptors plays a crucial role in regulating movement, motivation, locomotion, and working memory [157]. DA interacts with five different receptor subtypes, which are divided into two main groups: D1-like (including D1Rs and D5Rs) and D2-like (including D2Rs, D3Rs, and D4Rs) [158]. D1-like receptors are responsible for increasing the release of acetylcholine in the cortex, whereas D2-like receptors enhance cortical excitability. D2R receptors in the hippocampus correlate with memory function in AD, suggesting their role in cognitive aspects [159]. D3Rs, prevalent in DA-ergic neurons, regulate DA secretion, acting as auto-receptors and regulating DA secretion in ventral and dorsal striata [160]. D3Rs in mice inhibit DA release from presynaptic terminals; however, their contribution is significantly smaller compared with D2Rs [161, 162]. D4Rs, abundant in frontal lobe regions, modulate cognitive functions, whereas D5Rs are expressed postsynaptically in DA-stimulated cells of the hypothalamus [163].

In AD, existing data reveals elevated DA levels and a marked increase in DA receptors, particularly in the hippocampus and cortex [164, 165]. Animal models support these findings, showing reduced DA release in relevant brain regions. Biochemical, genetic, and animal model studies have also supported alterations of the DA-ergic system in AD. Both double mutant APP mice and 3xTg-AD mouse models of AD have reduced DA release in the hippocampus and insular cortex [166, 167]. Overall, cognitive impairment in AD may, at least in part, also be mediated through a deficit in DA-ergic transmission [168, 169]. The exact cause for the early occurrence of DA alterations in AD is not well understood. A study showed increased D2/3R levels in both the striatum and DA neuron cell body regions, coinciding with low amyloid and Tau deposition in the hippocampus [166]. While consensus on such changes in D2/3R density is lacking in humans [170, 171], reports of decreased DA synthesis in the AD brain imply that the observed increase in D2/3R density may be a compensatory response to the reduced availability of DA. Although the DA-ergic system is not a key player in AD, it is affected in AD [24, 172, 173], and no neuronal loss was observed before the formation of Aβ plaques. Belbin et al. linked the polymorphism of dopamine beta-hydroxylase with AD pathology [174]. Levels of DA and D1R and D2Rs were decreased in patients with AD in a recent meta-analysis linking the DA-ergic system and AD [175].

Human studies, utilizing PET and SPECT imaging, indicate decreased D1R and D2R, as well as DA transporters, in the nigrostriatal pathway in AD [149, 176–178], despite unaffected DA synthesis [179].

At the synapse, DA levels are regulated by the dopamine transporter (DAT), a transmembrane sodium-chloride-dependent protein selectively expressed on the presynaptic membrane of DA-ergic cells [180–182]. DAT is responsible for the reuptake of DA, terminating its activity in the synapse and modulating DA release while regulating its storage within synaptic vesicles via vesicular monoamine transporter 2 (VMAT2) [183, 184]. Specific VMAT2-binding radiopharmaceuticals for PET imaging of DA-ergic neurons allow differential diagnosis of Lewy body dementia from AD [185, 186].

In summary, the complex interactions among DA receptors, changes in levels, and modifications to the DA-ergic system contribute significantly to the cognitive impairment seen in AD. This underscores the importance of gaining a thorough understanding and developing specific therapeutic strategies tailored to address these neurochemical intricacies.

## Implications in Alzheimer’s neuropsychiatric treatment

In the face of escalating AD cases, current treatments focus on managing symptoms rather than halting or slowing the disease’s progression. While no cure exists for AD, various drug classes aim to enhance quality of life and functionality and manage psychiatric symptoms. However, these medications only temporarily suppress symptoms without addressing underlying pathology and may even worsen mortality, cognitive decline, and adverse events, raising concerns about cost-effectiveness. FDA-approved drug classes for AD include acetylcholinesterase inhibitors (e.g., donepezil, rivastigmine, galantamine), NMDA receptor antagonists (memantine), and more recently, monoclonal antibodies (mAbs) such as aducanumab (approved in 2021) and lecanemab (approved in 2023) (as shown in Table 2). These mAbs, along with others like bapineuzumab, gantenerumab, lecanemab, aducanumab, and donanemab, have shown mixed results in clinical trials. While aducanumab and lecanemab effectively reduce Aβ plaques, their cognitive benefits are modest and not clinically significant [187]. A review of 24 studies found that none of the mAbs, including FDA-approved lecanemab and aducanumab, showed cognitive improvements exceeding the minimal clinically important difference [188]. These drugs also come with significant side effects, potentially inducing inflammatory or hemorrhagic brain lesions in up to 40% of treated patients, and high costs [187, 189]. Other Aβ-targeting approaches, such as the γ-secretase inhibitor semagacestat and the BACE1 inhibitor verubecestat, reduced Aβ production but worsened cognitive decline, leading to early termination of trials [189]. Other mAbs, bapineuzumab and gantenerumab, have failed due to worsening cognitive decline or adverse events [189].

**Table 2:** Drugs targeting monoamines, amyloid beta, tau, and psychiatric symptoms aim to manage both cognitive decline and associated psychiatric symptoms in Alzheimer’s disease

Drug class	Examples	Mechanism	Potential effects	Limitations
**Selective serotonin reuptake inhibitors**	Fluoxetine, sertraline, citalopram	Inhibit the reuptake of serotonin, thereby increasing its levels in the synaptic cleft	May improve mood and reduce behavioral symptoms associated with AD	- Limited efficacy due to variability in response among individuals with Alzheimer’s disease - Adverse effects such as gastrointestinal issues or increased agitation in some patients
**Serotonin–norepinephrine reuptake inhibitors**	Venlafaxine, duloxetine	Inhibit the reuptake of both serotonin and norepinephrine, resulting in increased levels of these neurotransmitters	Similar to SSRIs, SNRIs may improve mood and alleviate behavioral symptoms	- Limited efficacy in treating severe depression or agitation - Adverse effects such as nausea, dizziness, or insomnia
**Dopamine agonists**	Pramipexole, ropinirole	Bind to dopamine receptors and mimic the action of dopamine	Improve cognitive function, particularly in domains related to attention and executive function	- Limited efficacy in addressing cognitive decline or neuropsychiatric symptoms in Alzheimer’s disease - Potential for adverse effects such as hallucinations, dizziness, or confusion
**Norepinephrine reuptake inhibitors**	Atomoxetine, Reboxetine	Atomoxetine inhibits the reuptake of norepinephrine, increasing its levels in the synaptic cleft. Reboxetine specifically targets norepinephrine reuptake	Atomoxetine may enhance attention and arousal, potentially improving cognitive function. Reboxetine may improve attention and cognitive function, which could potentially benefit individuals with AD	- Atomoxetine: Limited evidence in Alzheimer’s disease, potential for cardiovascular side effects - Reboxetine: Limited efficacy and potential for side effects such as dry mouth, constipation, and increased blood pressure
**Monoamine oxidase inhibitors**	Selegiline, rasagiline	Inhibit the activity of monoamine oxidase enzymes, which normally break down monoamine neurotransmitters	By increasing levels of monoamines such as serotonin, dopamine, and norepinephrine, MAOIs may have neuroprotective effects and improve cognitive function	- Potential for drug interactions and dietary restrictions (tyramine-containing foods), side effects such as hypertensive crisis with tyramine ingestion, and serotonin syndrome when combined with other serotonergic drugs
**Norepinephrine-dopamine reuptake inhibitors**	Bupropion	Inhibits the reuptake of both norepinephrine and dopamine	May improve mood and cognition by increasing levels of these neurotransmitters	- Limited efficacy in addressing mood symptoms such as depression or anxiety - Adverse effects such as insomnia, dry mouth, or agitation
**Serotonin modulators**	Trazodone, buspirone	Trazodone modulates serotonin activity through reuptake inhibition and receptor antagonism. Buspirone acts as a partial agonist at serotonin receptors	Trazodone may improve mood and reduce agitation. Buspirone may reduce anxiety and improve mood, potentially benefiting individuals with Alzheimer’s disease	- Trazodone: Limited evidence in Alzheimer’s disease, the potential for sedation, and orthostatic hypotension - Buspirone: Limited evidence in Alzheimer’s disease, potential for side effects such as dizziness and gastrointestinal disturbances
**Amyloid beta targeting drugs**	Aducanumab, solanezumab, gantenerumab	Target amyloid beta plaques, aiming to reduce their accumulation	May potentially slow disease progression by reducing the amyloid beta burden in the brain	- Aducanumab: Lack of consistent clinical benefit, side effects such as amyloid-related imaging abnormalities - Solanezumab/gantenerumab: Lack of significant clinical benefit, limited efficacy in later stages of Alzheimer’s disease
**Tau-targeting drugs**	ABBV-8E12, LMTM, TRx0237	Target hyperphosphorylated tau protein, aiming to reduce neurofibrillary tangle formation	May potentially slow cognitive decline by targeting tau pathology in Alzheimer’s disease	- Limited efficacy in clinical trials, difficulty in crossing the blood–brain barrier, and potential for adverse effects such as gastrointestinal disturbances and liver toxicity
**Antipsychotics**	Risperidone, quetiapine, olanzapine	Block dopamine receptors in the brain, reducing psychotic symptoms	May help manage psychotic symptoms such as hallucinations and delusions in individuals with Alzheimer’s disease	- Increased risk of stroke, mortality, or cognitive decline in elderly patients with dementia - Adverse effects such as sedation, extrapyramidal symptoms, or metabolic disturbances
	Aripiprazole, clozapine, haloperidol	Modulate dopamine and serotonin receptors, reducing agitation and aggression	May help manage agitation and aggression in individuals with Alzheimer’s disease	Side effects such as sedation, akathisia, and increased risk of stroke in elderly individuals
**Mood stabilizers**	Valproate, carbamazepine, lithium	Modulate neurotransmitter activity, stabilizing mood and reducing irritability	May help stabilize mood and reduce irritability in individuals with Alzheimer’s disease	- Risk of toxicity, particularly in elderly individuals, potential for cognitive impairment, weight gain, and metabolic disturbances
**Anxiolytics**	Lorazepam, alprazolam, diazepam	Enhance the effects of gamma-aminobutyric acid (GABA), reducing anxiety	May help alleviate anxiety symptoms in individuals with Alzheimer’s disease	- Risk of increased sedation, confusion, or falls in elderly patients - Potential for tolerance, dependence, or withdrawal symptoms with long-term use
**Antidepressants**	Mirtazapine, amitriptyline, escitalopram	Varying mechanisms, including serotonin and norepinephrine reuptake inhibition and serotonin receptor modulation	May improve mood and alleviate depressive symptoms in individuals with Alzheimer’s disease	- Side effects such as sedation, anticholinergic effects, and potential for drug interactions
**Psychostimulants**	Modafinil, methylphenidate, armodafinil	Increase dopamine and norepinephrine levels in the brain	May improve alertness, attention, and cognition in individuals with Alzheimer’s disease, potentially reducing apathy symptoms	Potential for cardiovascular side effects, insomnia

The recent approval of these mAbs marks a turning point in introducing disease-modifying properties, emphasizing the need to prioritize underlying pathology over symptomatic outcomes [187, 189]. However, current evidence suggests that the observed improvements from these mAbs fall far below what would be considered clinically meaningful, even with extended treatment durations. This paradigm shift poses challenges in integrating these drugs into clinical care, necessitating a clear understanding of their clinical impact, potential side effects, and establishment of clear stop criteria. Future research may need to focus on alternative strategies, better patient stratification, and exploring the potential of earlier treatment initiation.

There are over 200 candidate disease-modifying treatments for AD failing or being abandoned in clinical trials. These failures can be attributed to several factors, primarily stemming from an inadequate understanding of AD’s complex pathophysiology. Incorrect selection of main treatment targets, such as a singular focus on the amyloid hypothesis, and inappropriate drug dosages have been major issues. Additionally, initiating treatments in symptomatic AD patients may be too late, as the disease process likely begins years before cognitive decline manifests, necessitating presymptomatic or prodromal interventions [189].

The clinical trial methodology has also been criticized, particularly the lack of appropriate biomarkers for accurate patient selection, monitoring target engagement, and measuring treatment response [190]. In response to these limitations, clinical trials for AD have evolved with refined strategies aimed at overcoming past challenges and developing effective disease-modifying therapies. As a result, a significant shift has occurred in trial design, with a focus on earlier intervention and biomarker-based patient selection. Current trials–now target preclinical and prodromal populations aided by biomarkers like CSF amyloid-beta, total tau, and phosphorylated tau for early detection. Since most believe that AD needs to be treated years before the onset of symptoms, identifying early biomarkers of MA-ergic function is crucial. To effectively identify early biomarkers of MA-ergic function in AD, several advanced methods are utilized. CSF analysis of neurotransmitter metabolites, including 5-HT and DA byproducts, can provide early indicators of MA-ergic dysregulation [191–193]. PET with radiolabeled ligands targeting MA-ergic receptors offers detailed insights into receptor activity and distribution [194]. Neuroimaging techniques, such as MRI and functional MRI, detect structural and functional changes in MA-ergic pathways [195, 196]. Genetic and epigenetic analyses of variants affecting MA-ergic systems also aid in early risk assessment [197]. Recent advancements include blood-based assays for measuring peripheral neurotransmitter levels and metabolites, providing a less invasive monitoring approach [198, 199]. Integrating these methodologies enhances the capacity to detect and track MA-ergic dysfunction at the earliest stages of AD, facilitating the development of early intervention strategies [200]. Moreover, combination therapy regimens targeting multiple pathways are being evaluated, reflecting AD’s multifactorial nature. Novel candidate biomarkers spanning various pathways, including amyloid metabolism (CSF Aβ38, plasma BACE1), vascular dysregulation (CSF/serum heart-fatty acid binding protein), inflammation (CSF/blood TREM2, YKL-40), synaptic dysfunction (CSF neurogranin, SNAP-25, synaptotagmin), and others like CSF α-synuclein, plasma TDP-43, and iron metabolism markers, are being actively investigated for their potential utility in patient selection, stratification, and monitoring treatment response.

Despite recent FDA approvals for lecanemab and brexpirprazole, larger studies are needed to assess significant clinical improvement of NPS. No drugs are presently approved specifically for NPS in AD, aside from brexpirprazole for agitation, necessitating reliance on other interventions, though nonpharmacologic approaches often yield limited success. In cases where medications alleviate NPS, significant side effects may warrant continual risk–benefit analysis in treatment decisions.

Given the importance of MA-ergic systems in regulating mood, affective behaviors, and cognition, there is a longstanding interest in targeting these systems for psychiatric disorders. MA-ergic drugs have been used to control NPS in AD, however, with limited effectiveness. For example, the effectiveness of MAO-B inhibitors like selegiline remains controversial, potentially dependent on the DA neuron degeneration degree in the VTA, especially in early AD stages [24]. Similarly, efforts to target specific serotonin receptors for cognitive enhancement in AD have yielded mixed results, possibly due to protocol, task, and drug variability [105]. Research in animal models indicates that selective SSRIs might positively impact key AD-related biomarkers, such as amyloid and tau deposits, potentially delaying disease onset. SSRIs like citalopram and fluoxetine have shown efficacy in reducing amyloid burden and enhancing neurogenesis, whereas others, such as paroxetine, have demonstrated mixed or negative effects [111]. However, human studies present a mixed picture, with some indicating SSRIs’ potential to delay AD onset in individuals with prior depression, while others raise concerns about the risks associated with certain SSRIs [111]. The variability in study results highlights the need for more rigorous, long-term randomized controlled trials to determine the optimal use of SSRIs for AD prevention. Additionally, the emergence of new 5-HT-ergic antidepressants, such as vortioxetine, offers new avenues for research [111]. Multitarget drugs like vortioxetine [111, 201], influencing various serotonin components (5-HTR3, 5-HTR7, and 5-HTR1D, etc.), show promise in addressing cognitive impairments in AD by affecting multiple neurotransmitter systems critical for neural plasticity and cognitive processing [116].

Drugs targeting the noradrenergic system show promise in AD treatment, with evidence from animal studies and clinical trials indicating their potential to reduce inflammation and neurodegeneration. Noradrenaline reuptake inhibitors like desipramine, atomoxetine, and reboxetine have demonstrated anti-inflammatory effects by reducing pro-inflammatory cytokines, microglial activation markers, chemokines, and cell adhesion molecules in the brain cortex and hippocampus of animal models [202, 203]. Notably, reboxetine treatment in the 5xFAD transgenic mouse model decreased neuroinflammation, amyloid burden, and neurodegeneration [204]. Clinical trials with atomoxetine in mild cognitive impairment patients have shown encouraging results, normalizing cerebrospinal fluid biomarkers of tau, synaptic function, brain metabolism, and glial immunity while increasing brain activity in key circuits [205]. Monoamine oxidase inhibitors, though primarily developed for depression and Parkinson’s disease, have exhibited some benefits like improved cognition in AD animal models, though their clinical relevance remains unclear [204]. The noradrenaline precursor L-DOPS prevented microglial dysfunction, induced neurotrophic factors and amyloid-degrading enzymes, and mitigated memory impairments in 5xFAD mice [206, 207]. Vindeburnol, a compound that enhances tyrosine hydroxylase activity (the rate-limiting enzyme for noradrenaline synthesis), reduced amyloid plaque accumulation, induced BDNF expression, and prevented behavioral alterations in this AD mouse model [208]. Intriguingly, directly stimulating the LC, the principal noradrenergic nucleus, via vagus nerve stimulation, led to significant improvements in neuropsychological tests in a pilot clinical study of AD patients, with effects persisting after one year [209]. Chemogenetic activation of the locus coeruleus in transgenic rats also alleviated learning impairments [210].

Adrenergic receptors have also been investigated as potential targets for AD. Blockade of α2 adrenergic receptors not only effectively reduces Aβ load but also normalizes GSK3β hyperactivation and attenuates tau hyperphosphorylation [94, 95]. Moreover, such treatment improves cognitive functions in multiple AD animal models [94, 95, 211]. Prazosin, an α1-adrenergic receptor antagonist, can effectively mitigate agitation and aggressive manifestations in individuals with AD [104]. In APP23 mice, prazosin, not only ameliorated cognitive impairments but also bolstered the levels of anti-inflammatory cytokines [103]. β-Adrenergic receptor antagonists such as propranolol reduce Aβ accumulation in Tg2576 mice [101, 212, 213]. In SAMP8 mice, which exhibit accelerated aging, 3 weeks of propranolol treatment alleviated cognitive impairments and hyperphosphorylated tau levels [102]. Collectively, these findings from diverse preclinical models and early clinical studies highlight the immense therapeutic potential of targeting the noradrenergic system through various pharmacological and neurostimulation strategies to combat the multifaceted pathogenesis of AD [214].

## Conclusion

In 80–90% of AD cases, neuropsychiatric symptoms, ranging from mild (e.g., depression, anxiety) to severe (e.g., aggression, hallucinations), significantly impact both patient and caregivers, adding a substantial burden to the healthcare system. Understanding the molecular underpinnings of NPS in AD is essential for the development of effective treatment strategies to alleviate these devastating symptoms and improve patients’ quality of life.

Advances in neuroimaging, pathophysiological, and pharmacological studies reveal dysregulation of MA-ergic neurotransmission starting from the early stages of AD, which likely contributes to the NPS observed in AD. For example, DA deficits in the mesocortical-limbic pathway are linked to apathy and cognitive impairment in AD. Similarly, serotonin reductions contribute to depression, anxiety, and sleep disturbances. Noradrenergic neuron degeneration from the locus coeruleus leads to agitation, impulsivity, and cognitive deficits. The interplay between these neurotransmitter systems helps explain the overlap and co-occurrence of NPS in AD.

Research is shifting toward understanding individual variations, with precision medicine emerging as a promising avenue. By analyzing genetic and neurobiological profiles, precision medicine aims to tailor interventions, enhancing treatment effectiveness and minimizing adverse effects, ultimately paving the way for treatments that transcend the limitations of the monoamine-centric paradigm. Ongoing research aims to develop non-invasive biomarkers for early AD diagnosis, offering a timely and accurate alternative to invasive methods. Early detection allows for interventions that may alter the disease’s course and improve outcomes. In conclusion, the future of managing AD and its associated NPS lies in moving beyond the limitations of monoamine-based treatments. By embracing a deeper understanding of individual differences, pursuing the promise of precision medicine, and utilizing non-invasive biomarkers for early diagnosis, researchers aim to create a future where personalized interventions can significantly improve the quality of life for those living with this challenging condition.

While the understanding of AD and its NPS is evolving, there is optimism in exploring new avenues for managing NPS in AD. By investigating the molecular mechanisms of NPS, understanding the complexity of MA-ergic targets, and adopting personalized medicine strategies, more effective interventions may be possible. Additionally, the integration of non-invasive biomarkers for early detection could revolutionize AD management, offering timely interventions to potentially alter the disease’s course. Moving toward combined approaches including monoaminergic systems presents an opportunity for more holistic and impactful strategies in addressing AD and its associated NPS.

## Data Availability

All data supporting this review’s conclusions can be found in the cited references. This study summarizes existing research rather than generating new data, using only publicly available sources listed in the bibliography.

## Abbreviations

5-HT: Serotonin
5-HT-ergic: Serotonergic system
ACC: Anterior cingulate cortex
AD: Alzheimer’s disease
APP: Amyloid precursor protein
AβO: Amyloid-β oligomers
BPSD: Behavioral and Psychological Symptoms of Dementia
CSF: Cerebrospinal fluid
DA: Dopamine
DA-ergic: Dopaminergic system
EC: Entorhinal cortex
FDA: Food and Drug Administration
FDG-PET: Fluorodeoxyglucose positron emission tomography
LC: Locus coeruleus
MA-ergic: Monoaminergic system
MCI: Mild cognitive impairment
MRI: Magnetic resonance imaging
NA: Noradrenergic
NAcc: Nucleus accumbens
NE: Norepinephrine
NE-ergic: Norepinephrinergic system
NET: Norepinephrine transporter
NPI: Neuropsychiatric inventory
NPS: Neuropsychiatric symptoms
OFC: Orbitofrontal cortex
PFC: Prefrontal cortex
sMRI: Structural magnetic resonance imaging
SNc: Substantia nigra pars compacta
SORLA: Sortilin-related receptor with A-type repeats
SPECT: Single photon emission computed tomography
TH: Tyrosine hydroxylase
VMAT2: Vesicular monoamine transporter 2
VTA: Ventral tegmental area
α_2A_AR: Alpha-2A adrenergic receptor
